# Voltage Sensors Embedded in G Protein-Coupled Receptors

**DOI:** 10.3390/ijms25105295

**Published:** 2024-05-13

**Authors:** Merav Tauber, Yair Ben-Chaim

**Affiliations:** Department of Natural Sciences, The Open University of Israel, Ra’anana 4353701, Israel

**Keywords:** G protein coupled receptors, muscarinic receptor, voltage dependence, voltage sensor

## Abstract

Some signaling processes mediated by G protein-coupled receptors (GPCRs) are modulated by membrane potential. In recent years, increasing evidence that GPCRs are intrinsically voltage-dependent has accumulated. A recent publication challenged the view that voltage sensors are embedded in muscarinic receptors. Herein, we briefly discuss the evidence that supports the notion that GPCRs themselves are voltage-sensitive proteins and an alternative mechanism that suggests that voltage-gated sodium channels are the voltage-sensing molecules involved in such processes.

## 1. Introduction

G protein-coupled receptors (GPCRs) are the largest family of membrane proteins in the human body. Owing to their role in many cellular signaling processes and their pharmacological implications, they have been the focus of extensive research over the past few decades [1]. At present, the functions of these receptors—as well as the mechanisms of their activation and downstream signaling—are quite well understood at the molecular and, to some extent, structural levels. The activation of GPCRs usually starts with the binding of an extracellular agonist to a specific binding site on the receptor. This process stabilizes the receptor in its active conformation and allows it to activate its cognate G protein, as well as G protein-independent signaling pathways, such as signaling through β arrestin [2,3], which, in turn, triggers a variety of signaling processes within the cell.

Membrane potential is an important regulator of many cellular processes in various tissues and cell types. These processes include not only the excitability of neuronal cells, but also developmental processes such as cell migration, orientation, regeneration, and proliferation [4]. GPCRs are known to modulate membrane potential by activating ion channels. However, whether membrane potential can modulate GPCRs has not yet been considered. As we discuss below, several lines of evidence, both physiological and biochemical, have led to the notion that membrane potential is a novel regulatory modality of GPCRs.

## 2. Voltage Dependence of Muscarinic Receptors

The voltage dependence of GPCR-mediated signaling processes has been demonstrated in several systems. In a study, the binding of labeled acetylcholine (ACh) to muscarinic receptors in rat brain synaptosomes or synaptoneurosomes, as well as the interaction of muscarinic receptors with other proteins, was shown to be strong at resting potential and decrease when the membrane was depolarized by increasing KCl concentration [5,6,7,8]. In other studies, it was demonstrated that the ACh-induced Ca^2+^ response, mediated by muscarinic receptors in mouse pancreatic acinar cells, is sensitive to membrane potential [9,10]. Lastly, in another study, it was shown that M2R inhibits ACh release in a voltage-dependent manner [11].

**Figure 1:**
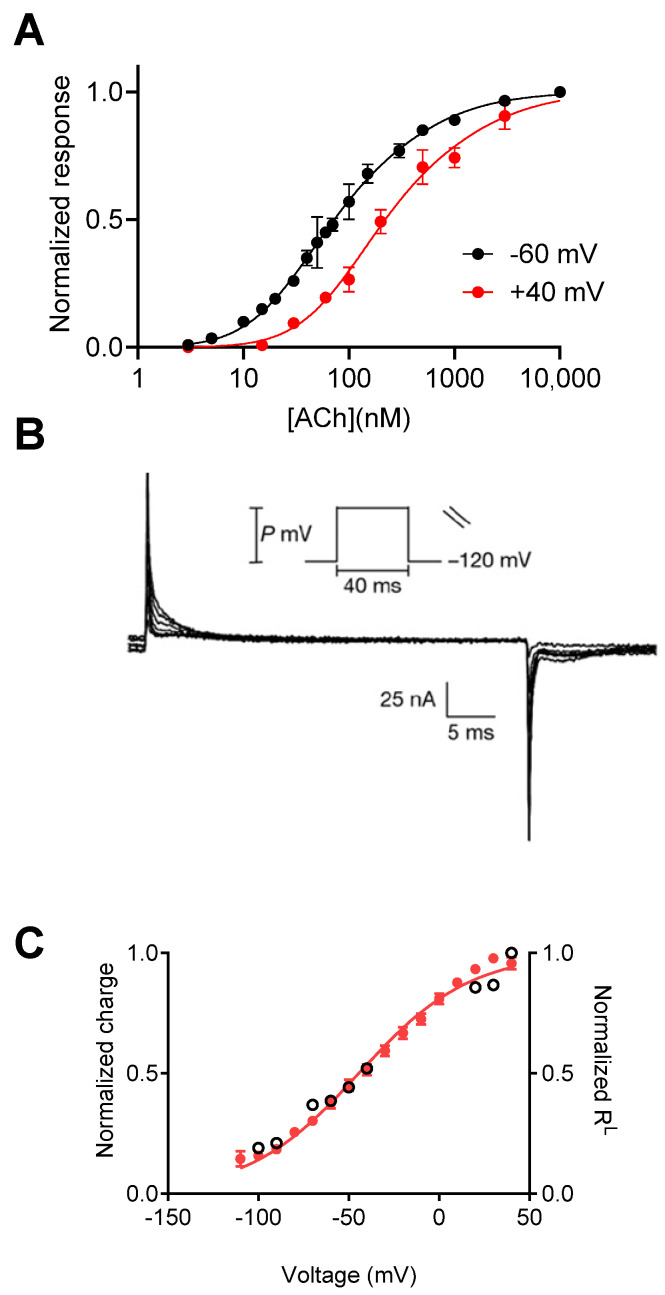
Voltage dependence of the M2R. (**A**) The dose–response curves obtained at −60 mV and +40 mV show a rightward shift under depolarization. (**B**) Charge movement-associated currents measured from M2R-expressing oocytes. (**C**) Current–voltage (red) and R^L^–voltage (empty circles) curves (receptors reside in a low-affinity state) showing a tight correlation between charge movement and shift in affinity. (**A**) is taken from [12]. (**B**,**C**) are taken from [16].

The studies mentioned above were all conducted in a physiological setting, and therefore, the exact mechanism of voltage dependence could not be attributed to the GPCR itself. Consequently, heterologous expression systems were utilized in order to elucidate the molecular basis for these voltage-dependent processes. The M2 muscarinic receptor (M2R), which mediates several of the aforementioned voltage-dependent processes, is a suitable candidate for such a study. To this end, the M2R was expressed in Xenopus oocytes, a well-established expression system for GPCRs and ion channels, together with the inward-rectifying G protein-activated potassium (GIRK) channel. In this functional system, M2R-activated GIRK currents were used as a measure of receptor activation. Electrophysiological measurements from these oocytes revealed that the apparent affinity of ACh and oxotremorine toward the M2R was modulated by membrane potential, as it was determined to be decreased upon depolarization (Figure 1A). Experiments involving the use of overexpressed βγ subunits and the non-hydrolyzable GTP analog (GTPγS) showed that this voltage dependence does not occur in downstream processes. Furthermore, measurement of the binding of labeled ACh to M2R-expressing oocytes at different membrane potentials directly showed that the binding of ACh to the receptor is voltage-dependent [12]. The results of a study showing that membrane potential affects the dissociation rate constant of the M2R further support the conclusion that the M2R itself is voltage-sensitive [13]. Similar approaches have been additionally used to examine whether the M1 muscarinic receptor (M1R) is voltage-sensitive as well. This receptor is a Gq-coupled receptor and therefore does not activate the GIRK channel. Instead, activation of the calcium-dependent chloride channels, endogenously expressed in Xenopus oocytes, was employed as a measure of receptor activation. Interestingly, it was found that membrane potential affected the binding of ACh to the M1R in an opposing manner, i.e., depolarization increased Ach’s potency and binding affinity toward the M1R rather than decreasing it.

A step forward in demonstrating that GPCRs are intrinsically voltage-sensitive came from the measurements of depolarization-induced charge movement-associated currents. These charge movements were first described in voltage-gated sodium channels, where they were interpreted as the movement of the gating charges that comprise the voltage sensor that leads to the opening of the channel pore, thus termed “gating currents” [14,15]. In GPCRs, gating currents were first measured using the cut-open voltage clamp configuration. As seen in Figure 1B, gating currents were observed to be M2R-expressing in response to depolarizing pulses. Similar currents were not detected in the control water-injected oocytes [16]. From these results, the dependence of the amount of charge that moves on membrane potential (Q-V) was measured, showing that the charge movement occurs in the physiological voltage range, with a V_1/2_ (voltage at half-maximal gating charge) of −44 mV. The slope of the Q-V curve, which is used to estimate the amount of charge that moves per receptor (although the interpretation of the slope value is not straightforward [17]), was found to be less than 1*e*_0_. This small value suggests weak voltage dependence of the M2R compared to voltage-gated channels (where the value equals ~12–14*e*_0_ moved per channel) [18]. Comparing the Q–V curve to the dependence of M2R affinity (the fraction of receptors in the low-affinity state, R^L^) on membrane potential showed that these two processes are correlated and thus possibly coupled (Figure 1C) [16].

Following these findings, Dekel et al. [19] designed additional experiments to investigate further the link between voltage and conformational changes in the M2R. In their study, they investigated whether depolarization induces a conformational change within the receptor protein. To answer this question, the authors labeled a specific residue near the orthosteric binding site of the M2R and simultaneously measured gating currents and voltage-dependent conformational changes using voltage-clamp fluorometry. They found that a conformational change occurs in the vicinity of the M2R binding site upon depolarization. The voltage dependence of this conformational change correlates well with both the voltage dependence of the gating currents and the voltage dependence of ACh affinity. Furthermore, treatments that eliminated the voltage-dependent shift in affinity, such as treatment with pertussis toxin (PTX), eliminated the voltage-dependent conformational changes, suggesting that these two processes are linked. Interestingly, PTX treatment did not affect the gating currents. This observation supports the view that the conformational change in the binding site is downstream of the depolarization-induced charge movements.

## 3. The Molecular Mechanism That Underlies Muscarinic Receptors’ Voltage Dependence

In voltage-dependent cellular processes, a protein or protein domain known as a ‘voltage sensor’ undergoes structural changes in response to shifts in membrane potential, thereby altering its function.

The notion that channels sense membrane potential was first proposed by Hodgkin and Huxley in their seminal work on action potential [20]. In the time since their study, ion channels have been characterized as voltage-dependent proteins. For such proteins, it has been demonstrated that changes in membrane potential induce a conformational change within the channel protein, thereby leading to channel opening and ions flowing through the channel pore [21,22,23]. The voltage sensor of these channels has been extensively studied over the past few decades at the molecular and structural levels. In particular, in determining the primary structure of sodium channels, the study results revealed that the fourth transmembrane segment (S4) contains a unique motif comprising four to seven repeated three-residue motifs of positively charged residues (usually arginine) followed by two hydrophobic residues [18]. Mutational analysis of this motif led to the suggestion that this motif serves as the voltage-sensing domain [22,23] and that changes in membrane potential result in the movement of these gating charges. This movement ultimately leads to a conformational change that changes the channel pore conformation, thus facilitating the opening and closing of the channel [23].

The voltage dependence of the M2R, and particularly the observation that depolarization induces charge movement in this receptor, suggests that this protein may contain a voltage-sensing motif. However, GPCRs, and specifically the M2R, do not contain a motif that is analogous to the canonical voltage sensor of ion channels, which makes the identification of the voltage sensor challenging.

**Figure 2:**
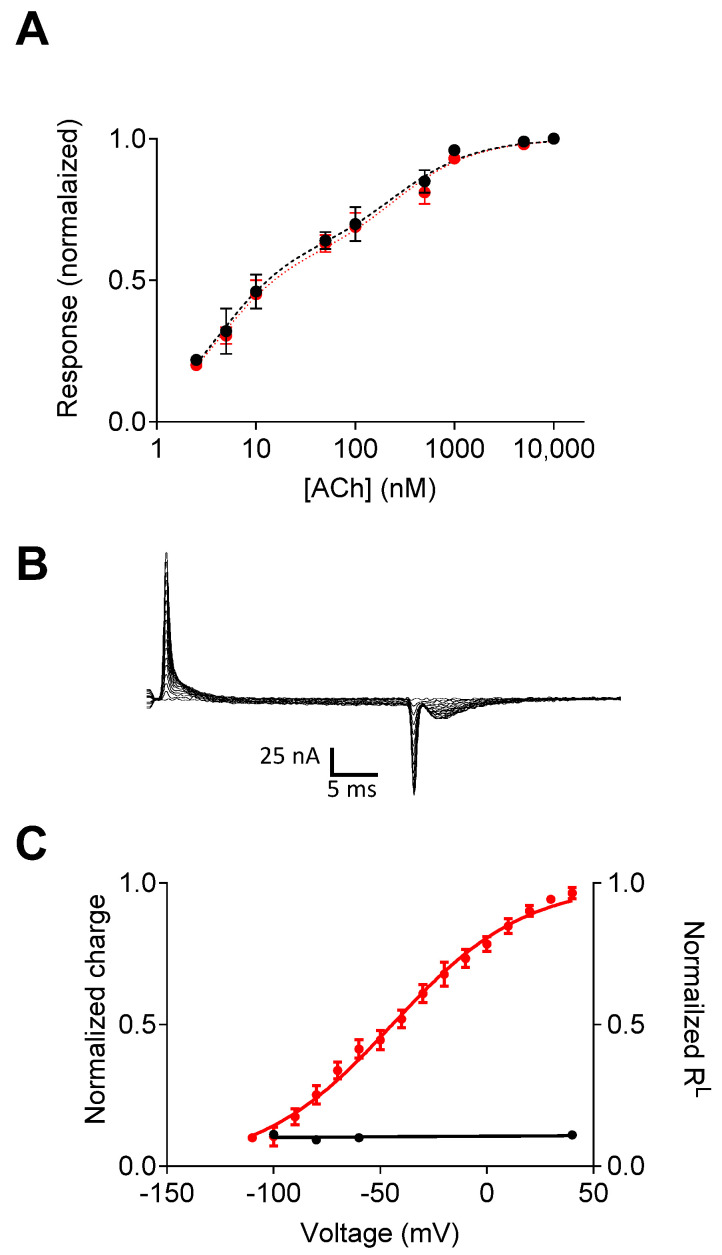
Voltage dependence of the M2R-ELAAL mutant. (**A**) The dose–response curves obtained at −60 mV (black) and +40 mV (red) indicate the elimination of the voltage dependence of agonist potency. (**B**) Charge movement-associated currents measured from M2R-ELAAL-expressing oocytes. (**C**) Current–voltage (red) and R^L^–voltage (black) curves. In the mutant receptor, the gating currents and the voltage dependence of affinity are not correlated. Figure taken from [16].

The author of a recent publication [24] suggested that muscarinic receptors might lack embedded voltage sensors. This suggestion was partly based on the attribution of the gating currents to charged residues (the KDKKE motif) in the N-terminal of the third intracellular loop of the receptor (L_3_), and the finding that removing these charges did not eliminate the gating currents (Figure 2A). However, the authors of this study did not conclude that these residues are indeed involved in the gating currents. Instead, it was suggested that the KDKKE motif plays a different role in the voltage dependence of the M2R. Specifically, replacing the KDKKE motif with ELAAL (the corresponding residues in the M1R) eliminated the voltage dependence of M2R binding affinity and the voltage dependence of Ach’s dissociation from the receptor [13]; this same alteration did not affect the charge movement-associated currents. As a result, the Q–V curve and R^L^-V curve measured from oocytes expressing this mutant were no longer correlated, as seen in Figure 2B. These observations led the authors to suggest that the KDKKE motif is probably not involved in voltage sensing, but that it may be essential for coupling the movement of the putative voltage sensor to changes in ligand-binding affinity in the M2R. The role of this motif in linking the gating currents to affinity change was further demonstrated by replacing the KDKKE motif with the ELAAL residues, which indeed eliminated the voltage-dependent conformational change while leaving the gating currents intact [16].

The observation that the M1R exhibits the opposite voltage dependence of affinity but shows similar gating currents is consistent with the idea that the voltage sensor might be coupled to affinity changes via L_3_. Namely, in the M1R, a similar voltage sensor is differentially coupled to changes in affinity (most likely via L_3_), thus resulting in different effects on the binding affinity of the receptor. This finding is in line with the finding that swapping the entire L_3_ between the M2R and M1R reversed the direction of the receptor’s voltage dependence. Following these findings, the authors further concluded that the coupling of voltage sensing to binding affinity probably involves other regions besides the N-terminal of L_3_ since the replacement of the KDKKE motif with ELAAL did not reverse the voltage dependence of the M2R.

The measurement of the voltage dependence of the three events, the gating currents, the conformational change in the binding site, and the binding affinity enabled the elucidation of the role of the KDKKE motif in the voltage-sensing process.

It is interesting to note that an analogous scenario occurs in voltage-gated ion channels. In these channels, positively charged residues within the S4 helix of the voltage-sensing domain are displaced in response to changes in voltage. This charge movement promotes a conformational change in the pore region. Similar to the opposite effect of depolarization on the affinity of the M1R and M2R, depolarization may either increase (e.g., in voltage-gated sodium or potassium channels) or decrease (in hyperpolarization-activated cyclic nucleotide-gated (HCN) channels) the open probability of the channels, although a similar voltage sensor is present in both types of voltage-gated channels.

The identity of the voltage-sensing motif(s) in the M2R has been further explored in other studies. In the study by Navarro-Polanco et al. [24], the authors replicated the gating current measurements in M2R-expressing oocytes and examined the effect of mutations in several residues of the M2R orthosteric binding site on the gating currents. They found that none of the reported mutations in the binding site affected the slope of the Q-V relation and are thus probably not part of the main voltage sensor. Some of these mutations had an effect on the V_1/2_ of the Q-V curve, however, suggesting that these residues play some form of indirect role in the movement of the voltage sensor [24], similar to the complex role of residues outside the S4-based voltage sensor in determining the voltage dependence of voltage-gated channels [25,26]. An interesting observation from the aforementioned study was that a mutation in the M2R Asp69^2.50^ residue eliminated the gating currents. However, this mutation also significantly reduced the expression of the receptor on the cell membrane, thus complicating the interpretation of this effect. The results of later studies showed that mutating this residue did not affect the voltage dependence of the affinity of the M2R [27], thus leaving the role of this residue in voltage sensing, for now at least, undetermined. In addition, in the study by Navarro-Polanco et al., the authors showed that both ACh and pilocarpine (a muscarinic partial agonist) affect the gating currents of the M2R, thus further supporting the conclusion that the M2R is the source of the measured gating currents [24].

**Figure 3:**
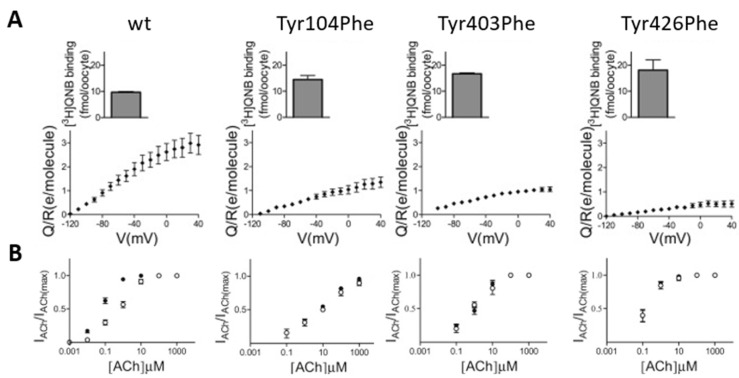
The effect of the “tyrosine lid” on voltage sensing in the M2R. (**A**) The dependence of the number of gating charges that move per receptor (Q/V) on membrane potential (V) for the wild-type M2R and the Tyr104Phe, Tyr403Phe, and Tyr426Phe mutants. The average expression level of each is shown in the insets. (**B**) The dose–response curves obtained from several experiments at −80 mV (solid circles) and +40 mV (open circles) using various concentrations of ACh from the wild-type M2R and the mutants, as indicated. Figure taken from [28].

Barchad-Avitzur et al. [28] employed a similar approach in their study in an attempt to identify the voltage sensor of the M2R. While charged residues are natural candidates to sense voltage, Navaro-Polanco already examined in their study the effect of neutralizing two negatively charged residues that are present in the transmembrane domains of the M2R (the aforementioned Asp69^2.50^ and the conserved Asp103^3.32^). Thus, the authors of this particular study examined the possibility that polar residues may act as voltage-sensing residues. Specifically, tyrosine has a strong intrinsic dipole moment [29], and it was therefore proposed that it might sense changes in membrane potential by reorienting its polar side chain [21]. The structure of muscarinic receptors indicated the existence of a conserved motif of three tyrosine residues (Tyr104^3.33^, Tyr403^6.51^, and Tyr426^7.39^; this numbering is for the M2R) that form a “lid” above the orthosteric ligand [30]. Hence, these tyrosine residues were proposed to affect ligand binding and receptor activation [30,31,32]. To examine the role of these residues in voltage sensing, each one of the three tyrosine residues was mutated to either alanine or phenylalanine, and gating currents were measured from oocytes expressing these mutants. We found that these mutations resulted in a decrease in the measured charge that moves per receptor (Figure 3A). Furthermore, these mutants exhibited a reduced voltage-induced conformational change. As a result, these mutations diminished the voltage dependence of M2R-induced GIRK currents (Figure 3B) [28]. These results suggest that these tyrosine residues form a voltage-sensing element in the M2R and perhaps in the M1R as well.

The findings of this study highlight the role of the orthosteric binding site in M2R voltage dependence. Rinne et al. [33] additionally showed in their study that the voltage dependence of other muscarinic receptors is dictated by the binding mode of agonists. They found that depolarization enhanced signaling by the M1R, as observed previously, but attenuated signaling by the M3R and M5R. Moreover, the effect of depolarization was also agonist-specific. For example, depolarization decreased M3R activation when the receptor was activated by ACh or carbachol but increased M3R activation when choline or pilocarpine was used.

Docking simulations corroborated these results by predicting that these ligands bind the receptors in two distinct binding modes and that these binding modes are associated with the effect of depolarization on receptor function. Mutation in the M3R following these predictions confirmed the correlation between binding mode and voltage dependence. A similar conclusion can be drawn from a recent study showing the correlation between the binding modes of μ opioid receptor agonists and their voltage sensitivity [34].

In addition to the orthosteric binding site, the allosteric binding site of the muscarinic receptors appears to play a role in the voltage dependence of these receptors as well. An initial clue for such involvement came from experiments showing that mutating two residues located at the allosteric binding site of the M2R (W422^7.35^ and W99^3.28^) eliminated voltage-dependent agonist affinity while leaving the gating currents intact [19]. Hoppe et al. [35] hypothesized and studied in detail the effects of alterations in the allosteric binding sites of M1 and M3 receptors on the voltage dependence of these receptors. To test their hypothesis, they constructed “allosteric site” chimeras of the M1R and M3R and analyzed their voltage dependencies. They found that exchanging all of the allosteric sites between the two receptors eliminated the voltage sensitivity of ACh responses for both receptors but did not affect their modulation by allosteric compounds. Furthermore, a point mutation in the M3R’s allosteric site eliminated the voltage dependence of the receptor and uncoupled the allosteric and orthosteric sites. The authors of this study further employed molecular dynamics simulations to demonstrate subtype-specific crosstalk between the two sites. Of note, the conserved tyrosine lid structure of the orthosteric site, mentioned above as a putative voltage-sensing motif, was suggested to take part in this crosstalk. They concluded that the allosteric binding site plays a role in determining the subtype-specific voltage dependence of muscarinic receptors.

The accumulating evidence presented above, showing the direct effects of receptor alteration on the voltage dependence of their gating currents and activation, strongly supports the conclusion that the receptor itself is responsible for the gating currents and voltage-dependent agonist binding.

## 4. Alternative Mechanism for Muscarinic Receptors’ Voltage Dependence

A recent publication by Cohen-Armon challenges the conclusion that muscarinic receptors are intrinsically voltage-dependent. This publication proposes an alternative mechanism to account for the voltage dependence of muscarinic receptors. The proposed mechanism does not assume the intrinsic voltage dependence of the receptor [36]. This proposal originated from in vitro experiments suggesting that agonist binding to muscarinic receptors and Na^+^ channels is coupled via G protein [37,38]. Following these studies, other studies were performed in synaptoneurosomes, a preparation that consists of resealed presynaptic terminals and postsynaptic neurons and thus contains the native environment of a synapse. In these studies, it was further shown that muscarinic agonists induced the uptake of sodium ions into the synaptoneurosomes; in contrast, the muscarinic antagonist atropine blocked sodium uptake. The results of additional experiments suggested that the depolarization-induced opening of sodium channels is essential for the depolarization-induced activation of G_o_-proteins, which modulate the affinity of muscarinic receptors, shifting them into a low-affinity state [39,40]. According to the proposed mechanism, muscarinic receptors interact with voltage-gated sodium channels, and depolarization-induced opening of these channels promotes the activation of G proteins, which may affect, in turn, muscarinic agonist affinity. However, the results of the experiments that led to the proposed mechanism may not be conclusive as the majority of them were conducted in synaptoneurosomes, where other channels, receptors, and signaling molecules are expressed. Thus, it is difficult to identify the voltage-sensing molecule. In addition, the experiments largely relied on pharmacological means, which may not be specific. For example, the compound diphenyleneiodonium (DPI) was used to prevent the opening of voltage-gated sodium channels. This compound has been shown to act on other targets as well [41]. Of note, it has been shown to affect directly muscarinic receptors [42]. It is thus possible that the suggested effect of sodium channels on muscarinic receptors in these experiments actually reflects other cellular processes. These findings may be linked to the known allosteric effect of sodium ions on class A GPCRs [43,44]. We have recently shown that the M2R is indeed modulated by extracellular sodium ions [45]. This modulatory effect of sodium ions was proposed to play a role in the voltage dependence of GPCRs [46,47].

While the mechanism proposed above may be feasible in brain synaptoneurosomes, it is unlikely that it is responsible for the voltage dependence of muscarinic receptors observed in other systems. Voltage-gated sodium channels are not expressed in significant amounts in Xenopus oocytes, in which many of the experiments concerning the voltage dependence of GPCRs, including the recordings of gating currents, were conducted. Furthermore, ionic currents and gating currents have been measured in numerous studies employing Xenopus oocytes. To the best of our knowledge, none of these studies has reported the presence of such channels in any significant amount, and gating currents are not observed in water-injected oocytes. Furthermore, ionic currents and gating currents have been measured in numerous studies employing Xenopus oocytes. Gating currents from the M2R and M1R were recorded from oocytes where no other proteins were exogenously expressed and under conditions designed to eliminate any ionic currents. Moreover, similar voltage dependence of muscarinic receptors has been observed in other cell types, such as atrial cells [24] and HEK293 cells [33]. It is unlikely that the same mechanism observed in synaptoneurosomes occurs in these cell types, which may express a different set of ion channels. Furthermore, additional lines of evidence directly link the muscarinic receptor protein to the voltage dependence observed. First, as mentioned above, the kinetics of the gating currents, as well as their voltage dependence and the gating charge amplitude, were altered by mutating specific residues within the receptor [24,28]. Second, in some cases, mutations in the M1R, M2R, and M3R affected the voltage dependence of both conformational changes in the binding site and the agonist binding affinity [24,28,33,35]. Finally, point mutations in M3R altered the effect of voltage on the activation of the receptor measured using FRET [35]. Another observation that may be relevant to this discussion is that the voltage dependence of muscarinic receptors has been shown to be agonist-dependent as well, i.e., while depolarization may decrease the potency of some ligands toward receptors, it may have no effect or even the opposite effect on other ligands [24,33,48]. Such ligand-specific voltage dependence has been observed for several other GPCRs [34,49,50,51,52]. Furthermore, point mutations within the receptor protein have been shown to change this ligand specificity [33,34].

Taken together, the cumulative experimental findings strongly suggest that GPCRs themselves are voltage-sensitive. This voltage dependence is likely achieved by the depolarization-induced charge movement that results in a conformational change that affects receptor affinity and/or potency.

These findings seem inconsistent with the suggestion that voltage-gated sodium channels are indispensable in this process.

## 5. Physiological Implications of Voltage Dependence of Muscarinic Receptors and Other GPCRs

In conclusion, it is worth noting that since the identification of muscarinic receptor voltage dependence, several other GPCRs, including receptors to glutamate [53,54], dopamine [50,55,56,57], adrenaline [58,59], histamine [60], serotonin [51], opioids [49], prostanoids [61], and cannabinoids [52], have been shown to be voltage-dependent. This wide array of voltage-dependent GPCRs suggests that membrane potential may serve as an important and universal modulator of cell-signaling processes via GPCRs. Indeed, the physiological role of voltage-dependent GPCRs is starting to be revealed in various processes. In atrial cells, potassium currents that are activated by the M2R show relaxation, i.e., a slow decrease in current amplitude with depolarization. The nature of this behavior was previously unknown. Several studies conducted following the discovery of the voltage dependence of the M2R suggest that this property of the M2R is responsible for this behavior. Namely, depolarization reduces the affinity of the receptor, and thus, the potassium currents are inactivated [48,62,63]. Another well-studied example is the role of the M2R in the control of neurotransmitter release. Based on other experimental data and theoretical considerations, the Ca^2+^ voltage hypothesis for neurotransmitter release was proposed [64,65,66]. According to this hypothesis, the action potential that depolarizes the presynaptic terminal decreases the affinity of the autoreceptor and thereby weakens the interaction of the receptor with the synaptic proteins of the release machinery. The free-release machinery can then, in turn, promote transmitter release [65]. Indeed, it has been shown that the M2R interacts with the protein of the release machinery in a voltage-dependent manner [7,8]. More direct evidence to support this hypothesis has been obtained through the use of mouse neuromuscular synapses [11]. In this particular study, inhibition of the gating currents in the M2R through the rapid application of muscarinic ligands modified the time course and degree of transmitter release, suggesting a direct, Ca^+2^-independent role for membrane potential in controlling this fundamental neuronal process via the M2R. Similarly, the results of recent reports have indicated a role for voltage-dependent metabotropic glutamate receptors in controlling neurotransmitter release [54,67]. Finally, the results of a recent study have demonstrated, for the first time, a role for GPCRs’ voltage-dependent behavior. Specifically, it was demonstrated that the type A muscarinic receptor of *Drosophila melanogaster* exhibits voltage dependence, similar to the mammalian M1R, and that mutating residues in a location that corresponds to the ELAAL motif eliminated its voltage dependence. Electrophysiological and behavioral experiments conducted in a fly strain that carries the same mutation showed impaired learning behavior. These results suggest that the voltage dependence of this receptor is crucial for this behavior in the relevant fly [68]. Together, these physiological findings suggest that the voltage dependence of GPCRs is a general phenomenon, and is relevant to various signaling processes and brain functions.

The studies of Cohen-Armon and colleagues were among the first to demonstrate voltage dependence in processes mediated by GPCRs and led the way for other discoveries in the field. These discoveries shed some light on the generality of this phenomenon, its physiological implications, and the underlying molecular mechanism [69,70]. The results from several laboratories studying various GPCRs have revealed a novel way through which membrane proteins sense voltage. The voltage dependence of GPCRs emerges as a multifaceted phenomenon likely involving the interplay of multiple receptor modalities. However, a comprehensive understanding of this intricate process remains elusive. The putative link between the sodium channels and muscarinic receptor-mediated voltage-dependent processes studied by Cohen-Armon and colleagues should be revisited in light of the current evidence.

## References

[1] Hauser A.S., Attwood M.M., Rask-Andersen M., Schiöth H.B., Gloriam D.E. (2017). Trends in GPCR drug discovery: New agents, targets and indications. Nat. Rev. Drug Discov..

[2] Gurevich V.V., Gurevich E.V. (2019). GPCR Signaling Regulation: The Role of GRKs and Arrestins. Front. Pharmacol..

[3] Wang W., Qiao Y., Li Z. (2018). New Insights into Modes of GPCR Activation. Trends Pharmacol. Sci..

[4] Kadir L.A., Stacey M., Barrett-Jolley R. (2018). Emerging Roles of the Membrane Potential: Action Beyond the Action Potential. Front. Physiol..

[5] Cohen-Armon M., Sokolovsky M. (1991). Depolarization-induced changes in the muscarinic receptor in rat brain and heart are mediated by pertussis-toxin-sensitive G-proteins. J. Biol. Chem..

[6] Cohen-Armon M., Garty H., Sokolovsky M. (1988). G-protein mediates voltage regulation of agonist binding to muscarinic receptors: Effects on receptor-sodium channel interaction. Biochemistry.

[7] Linial M., Ilouz N., Parnas H. (1997). Voltage-Dependent Interaction Between the Muscarinic ACh Receptor and Proteins of the Exocytic Machinery. J. Physiol..

[8] Ilouz N., Branski L., Parnis J., Parnas H., Linial M. (1999). Depolarization Affects the Binding Properties of Muscarinic Acetylcholine Receptors and Their Interaction with Proteins of the Exocytic Apparatus. J. Biol. Chem..

[9] Marty A., Tan Y.P. (1989). The initiation of calcium release following muscarinic stimulation in rat lacrimal glands. J. Physiol..

[10] Ong B.H., Ohsaga A., Sato K., Oshiro T., Shirato K., Maruyama Y. (2001). G protein modulation of voltage-sensitive muscarinic receptor signalling in mouse pancreatic acinar cells. Pflügers Archiv.

[11] Kupchik Y.M., Barchad-Avitzur O., Wess J., Ben-Chaim Y., Parnas I., Parnas H. (2011). A novel fast mechanism for GPCR-mediated signal transduction—Control of neurotransmitter release. J. Cell Biol..

[12] Ben-Chaim Y., Tour O., Dascal N., Parnas I., Parnas H. (2003). The M2 muscarinic G-protein-coupled receptor is voltage-sensitive. J. Biol. Chem..

[13] Chaim Y.B., Bochnik S., Parnas I., Parnas H. (2013). Voltage Affects the Dissociation Rate Constant of the m2 Muscarinic Receptor. PLoS ONE.

[14] Armstrong C.M., Bezanilla F. (1973). Currents Related to Movement of the Gating Particles of the Sodium Channels. Nature.

[15] Bezanilla F. (2018). Gating currents. J. Gen. Physiol..

[16] Ben-Chaim Y., Chanda B., Dascal N., Bezanilla F., Parnas I., Parnas H. (2006). Movement of “gating charge” is coupled to ligand binding in a G-protein-coupled receptor. Nature.

[17] Bezanilla F., Villalba-Galea C.A. (2013). The gating charge should not be estimated by fitting a two-state model to a Q-V curve. J. Gen. Physiol..

[18] Catterall W.A. (2010). Ion channel voltage sensors: Structure, function, and pathophysiology. Neuron.

[19] Dekel N., Priest M.F., Parnas H., Parnas I., Bezanilla F. (2012). Depolarization induces a conformational change in the binding site region of the M_2_ muscarinic receptor. Proc. Natl. Acad. Sci. USA.

[20] Hodgkin A.L., Huxley A.F. (1952). A quantitative description of membrane current and its application to conduction and excitation in nerve. J. Physiol..

[21] Bezanilla F. (2008). How membrane proteins sense voltage. Nat. Rev. Mol. Cell Biol..

[22] Bezanilla F. (2000). The Voltage Sensor in Voltage-Dependent Ion Channels. Physiol. Rev..

[23] Bezanilla F., Perozo E. (2003). The voltage sensor and the gate in ion channels. Advances in Protein Chemistry.

[24] Navarro-Polanco R.A., Galindo E.G.M., Ferrer-Villada T., Arias M., Rigby J.R., Sánchez-Chapula J.A., Tristani-Firouzi M. (2011). Conformational changes in the M2 muscarinic receptor induced by membrane voltage and agonist binding. J. Physiol..

[25] Zhang L., Wu X., Cao X., Rao K., Hong L. (2024). Trp207 regulation of voltage-dependent activation of human Hv1 proton channel. J. Biol. Chem..

[26] Ma Z., Lou X.J., Horrigan F.T. (2006). Role of charged residues in the S1-S4 voltage sensor of BK channels. J. Gen. Physiol..

[27] Ågren R., Sahlholm K., Nilsson J., Århem P. (2018). Point mutation of a conserved aspartate, D69, in the muscarinic M2 receptor does not modify voltage-sensitive agonist potency. Biochem. Biophys. Res. Commun..

[28] Barchad-Avitzur O., Priest M.F., Dekel N., Bezanilla F., Parnas H., Ben-Chaim Y. (2018). A Novel Voltage Sensor in the Orthosteric Binding Site of the M2 Muscarinic Receptor. Biophys. J..

[29] Momany F.A., McGuire R.F., Burgess A.W., Scheraga H.A. (1975). Energy parameters in polypeptides. VII. Geometric parameters, partial atomic charges, nonbonded interactions, hydrogen bond interactions, and intrinsic torsional potentials for the naturally occurring amino acids. J. Phys. Chem..

[30] Kruse A.C., Ring A.M., Manglik A., Hu J., Hu K., Eitel K., Hübner H., Pardon E., Valant C., Sexton P.M. (2013). Activation and allosteric modulation of a muscarinic acetylcholine receptor. Nature.

[31] Gregory K.J., Hall N.E., Tobin A.B., Sexton P.M., Christopoulos A. (2010). Identification of orthosteric and allosteric site mutations in M2 muscarinic acetylcholine receptors that contribute to ligand-selective signaling bias. J. Biol. Chem..

[32] DeVree B.T., Mahoney J.P., Vélez-Ruiz G.A., Rasmussen S.G.F., Kuszak A.J., Edwald E., Fung J.-J., Manglik A., Masureel M., Du Y. (2016). Allosteric coupling from G protein to the agonist-binding pocket in GPCRs. Nature.

[33] Rinne A., Mobarec J.C., Mahaut-Smith M., Kolb P., Bünemann M. (2015). The mode of agonist binding to a G protein–coupled receptor switches the effect that voltage changes have on signaling. Sci. Signal.

[34] Kirchhofer S.B., Lim V.J.Y., Ernst S., Karsai N., Ruland J.G., Canals M., Kolb P., Bünemann M. (2023). Differential interaction patterns of opioid analgesics with µ opioid receptors correlate with ligand-specific voltage sensitivity. Elife.

[35] Hoppe A., Marti-Solano M., Drabek M., Bünemann M., Kolb P., Rinne A. (2018). The allosteric site regulates the voltage sensitivity of muscarinic receptors. Cell Signal.

[36] Cohen-Armon M. (2023). Are Voltage Sensors Really Embedded in Muscarinic Receptors?. Int. J. Mol. Sci..

[37] Cohen-Armon M., Sokolovsky M. (1986). Interactions between the muscarinic receptors, sodium channels, and guanine nucleotide-binding protein(s) in rat atria. J. Biol. Chem..

[38] Cohen-Armon M., Kloog Y., Henis Y.I., Sokolovsky M. (1985). Batrachotoxin changes the properties of the muscarinic receptor in rat brain and heart: Possible interaction(s) between muscarinic receptors and sodium channels. Proc. Natl. Acad. Sci. USA.

[39] Anis Y., Nürnberg B., Visochek L., Reiss N., Naor Z., Cohen-Armon M. (1999). Activation of Go-proteins by Membrane Depolarization Traced by in Situ Photoaffinity Labeling of Gαo-proteins with [α32P]GTP-azidoanilide*. J. Biol. Chem..

[40] Cohen-Armon M., Sokolovsky M. (1993). Evidence for involvement of the voltage-dependent Na+ channel gating in depolarization-induced activation of G-proteins. J. Biol. Chem..

[41] Siegl P.K.S., Garcia M.L., King V.F., Scott A.L., Morgan G., Kaczorowski G.J. (1988). Interactions of DPI 201-106, a novel cardiotonic agent, with cardiac calcium channels. Naunyn Schmiedebergs Arch. Pharmacol..

[42] Groschner K., Ulle P., Brunner F., Kukovetz W.R. (1989). Interaction of DPI 201-106 with cardiac muscarinic receptors. Eur. J. Pharmacol..

[43] Katritch V., Fenalti G., Abola E.E., Roth B.L., Cherezov V., Stevens R.C. (2014). Allosteric sodium in class A GPCR signaling. Trends Biochem. Sci..

[44] Liu W., Chun E., Thompson A.A., Chubukov P., Xu F., Katritch V., Han G.W., Roth C.B., Heitman L.H., IJzerman A.P. (2012). Structural basis for allosteric regulation of GPCRs by sodium ions. Science.

[45] Friedman S., Tauber M., Ben-chaim Y. (2020). Sodium ions allosterically modulate the M2 muscarinic receptor. Sci. Rep..

[46] Vickery O.N., Machtens J.-P., Zachariae U. (2016). Membrane potentials regulating GPCRs: Insights from experiments and molecular dynamics simulations. Curr. Opin. Pharmacol..

[47] Vickery O.N., Machtens J.-P., Tamburrino G., Seeliger D., Zachariae U. (2016). Structural Mechanisms of Voltage Sensing in G Protein-Coupled Receptors. Structure.

[48] Moreno-Galindo E.G., Alamilla J., Sanchez-Chapula J.A., Tristani-Firouzi M., Navarro-Polanco R.A. (2016). The agonist-specific voltage dependence of M2 muscarinic receptors modulates the deactivation of the acetylcholine-gated K^+^ current *I*_KACh_). Pflugers Arch..

[49] Ruland J.G., Kirchhofer S.B., Klindert S., Bailey C.P., Bünemann M. (2020). Voltage modulates the effect of μ-receptor activation in a ligand-dependent manner. Br. J. Pharmacol..

[50] Sahlholm K., Marcellino D., Nilsson J., Fuxe K., Århem P. (2008). Voltage-sensitivity at the human dopamine D2S receptor is agonist-specific. Biochem. Biophys. Res. Commun..

[51] Tauber M., Chaim Y.B. (2022). The activity of the serotonergic 5-HT1A receptor is modulated by voltage and sodium levels. J. Biol. Chem..

[52] Goldberger E., Tauber M., Ben-Chaim Y. (2022). Voltage dependence of the cannabinoid CB1 receptor. Front. Pharmacol..

[53] Ohana L., Barchad O., Parnas I., Parnas H. (2006). The Metabotropic Glutamate G-protein-coupled Receptors mGluR3 and mGluR1a Are Voltage-sensitive. J. Biol. Chem..

[54] Boutonnet M., Carpena C., Bouquier N., Chastagnier Y., Font-Ingles J., Moutin E., Tricoire L., Chemin J., Perroy J. (2024). Voltage tunes mGlu_5_ receptor function, impacting synaptic transmission. Br. J. Pharmacol..

[55] Ågren R., Sahlholm K. (2020). Voltage-Dependent Dopamine Potency at D1-Like Dopamine Receptors. Front. Pharmacol..

[56] Sahlholm K., Nilsson J., Marcellino D., Fuxe K., Århem P. (2008). Voltage-dependence of the human dopamine D2 receptor. Synapse.

[57] Sahlholm K., Marcellino D., Nilsson J., Fuxe K., Århem P. (2008). Differential voltage-sensitivity of D2-like dopamine receptors. Biochem. Biophys. Res. Commun..

[58] Birk A., Rinne A., Bünemann M. (2015). Membrane Potential Controls the Efficacy of Catecholamine-induced β1-Adrenoceptor Activity. J. Biol. Chem..

[59] Rinne A., Birk A., Bünemann M. (2013). Voltage regulates adrenergic receptor function. Proc. Natl. Acad. Sci. USA.

[60] Sahlholm K., Nilsson J., Marcellino D., Fuxe K., Århem P. (2012). Voltage sensitivities and deactivation kinetics of histamine H3 and H4 receptors. Biochim. Et Biophys. Acta Biomembr..

[61] Kurz M., Krett A.-L., Bünemann M. (2020). Voltage Dependence of Prostanoid Receptors. Mol. Pharmacol..

[62] Moreno-Galindo E.G., Sánchez-Chapula J.A., Sachse F.B., Rodríguez-Paredes J.A., Tristani-Firouzi M., Navarro-Polanco R.A. (2011). Relaxation gating of the acetylcholine-activated inward rectifier K^+^ current is mediated by intrinsic voltage sensitivity of the muscarinic receptor. J. Physiol..

[63] Salazar-Fajardo P.D., Aréchiga-Figueroa I.A., López-Serrano A.L., Rodriguez-Elias J.C., Alamilla J., Sánchez-Chapula J.A., Tristani-Firouzi M., Navarro-Polanco R.A., Moreno-Galindo E.G. (2018). The voltage-sensitive cardiac M2 muscarinic receptor modulates the inward rectification of the G protein-coupled, ACh-gated K^+^ current. Pflugers Arch..

[64] Parnas H., Parnas I. (2007). The chemical synapse goes electric: Ca^2+^- and voltage-sensitive GPCRs control neurotransmitter release. Trends Neurosci..

[65] Parnas I., Parnas H. (2010). Control of neurotransmitter release: From Ca^2+^ to voltage dependent G-protein coupled receptors. Pflugers Arch..

[66] Parnas H., Segel L., Dudel J., Parnas I. (2000). Autoreceptors, membrane potential and the regulation of transmitter release. Trends Neurosci..

[67] Zhang Q., Liu B., Li Y., Yin L., Younus M., Jiang X., Lin Z., Sun X., Huang R., Liu B. (2020). Regulating quantal size of neurotransmitter release through a GPCR voltage sensor. Proc. Natl. Acad. Sci. USA.

[68] Rozenfeld E., Tauber M., Ben-Chaim Y., Parnas M. (2021). GPCR voltage dependence controls neuronal plasticity and behavior. Nat. Commun..

[69] Mahaut-Smith M.P., Martinez-Pinna J., Gurung I.S. (2008). A role for membrane potential in regulating GPCRs?. Trends Pharmacol. Sci..

[70] David D., Bentulila Z., Tauber M., Ben-Chaim Y. (2022). G Protein-Coupled Receptors Regulated by Membrane Potential. Int. J. Mol. Sci..

